# Supplementary material for: Elite athletes’ lifestyles: Consumerism to professionalism
Source: PLoS One. 2022 Sep 26;17(9):e0269287. doi: 10.1371/journal.pone.0269287 (PMC9512170; doi:10.1371/journal.pone.0269287)
Supplement: S1 File — (DOCX) [file pone.0269287.s001.docx]

|  | ***Excerpts of the transcripts*** |
| --- | --- |
| ***1*** | *…, my life as an athlete is of interest to my fans, the media, and even everyone. I think I have to be different from everyone else and show that distinction in different ways…,*  *I always like to try anything new that comes on the market before anyone else, now that new thing can be a new car, a new phone, a new hobby, and not anything, that ordinary people can easily afford it. Of course, there may be very rich people who use unique and rare things, but I, as an athlete, get a lot more attention. The things I said are appearance, and even in many cases, living like this is what my fans expect. I mean, a lot of them are in favor of me because they think I'm special and I have to show that I'm special…,*  *…, Although it can sometimes be difficult to make ends meet, the very special life that appeals to many makes me more of a fan of fashion and clothing companies. I have different relationships and it all makes me feel good. The next point is that living like these costs a lot, but I think I only have the conditions to experience this particular life once and I have to make the most of it; I'm an athlete now, and I make good money so I can use whatever I want. If the situation changes for me later, then I will think differently…,*  *…, I have already said that this is the appearance of life that I would like others to know about, and even if the media show my pictures with a special type and fashion, new clothes, modern accessories, luxury cars, very expensive brands, etc. There is no problem. But it cannot always be allowed, especially in our country; Let everyone see all parts of a person's life, so I will never, ever expose a part of my life to the public, and only very special and close people can find out about it. Now, you may be curious and guess I mean special relationships. Of course, you are right, but I will not say more about that…,* |
| ***2*** | *Football has become a means for people to know me and I am happy about that, but I have always asked God to put a path in front of my feet so that people will love me not as a footballer but because of my social work. I go to people because of my social personality, my activities, and my thoughts, and I think it has happened now. Cups are things that take you to the heart of history, but social work makes you feel good for a few weeks; I want this good mood. But I apologize to all those who ask me for help; I'm a person with limited assets…, The requests from me are so many that if I sell my whole property, I will not be able to respond to the requests for more than two days, and this is a great pain that is always in my chest. Because I have always asked God to make all the people of my country prosperous…,*  *…The media have written that I live in a penthouse of 1,400 meters and 2,000 meters. First, I have to say that with all the effort I put in, I have to have a prosperous life because I think it is my right, but I do not live in a penthouse. My house is 418 meters. If I wanted to live differently, I might have had 20 to 20-story towers, but I always like to live healthily. Because living healthy also guarantees my baby's future…,* |
| ***3*** | *…. Honestly, high-level exercise is very hard and I have to train very seriously as an athlete to be able to perform well. So, if I divide my life into two parts, the sports and non-sports section, I would like the non-sports section to be completely comfortable and relaxed. That is, if I have difficulty in the sports section of my life, I want to be comfortable in the non-sports section, and spend my time without thinking about a specific program. I mean, I do not see the need to make precise decisions to choose my work and I make decisions in every situation, in many cases, I do not even bother to make decisions and I easily accept the suggestions of my friends and those around me. Many people like me like to think carefully and plan for their free time, but I do not need this and why should I put myself in trouble in my free time at all. I like to spend my free time at home with comfortable and effortless activities. This is better because I do not have to expose myself to the public and later answer numerous questions from coaches, people around me, and even the media. Some may say that I live this way out of laziness, but now I like living this way, I may choose another way of living later and in other circumstances. That is, I do not have to accept everything that is accepted and adapt myself to the minds of others…* |
| ***4*** | *…. the most important thing in my life decisions is the society or community in which I find myself. I mean, even being an athlete is because there is a community that cares about sports, and because sports are valuable in the community, I have become valuable as an athlete. So, I think society should be the most important factor that I have to consider in my life. Reputation and fame make me more sensitive to society because members of society expect me not to be indifferent to their problems, that is, they expect me to have positive effects, and I try, to the extent I may show my role as a good athlete. Now the situation is such that some people like to expose themselves to the public and be noticed in any way possible, but this thinking can work to some extent and people end up with an athlete who considers himself superior to them. See, they will be disgusted. But if an athlete is useful to society, or at least tries to prove himself useful, it will make the athlete respected and become a very popular person. If an athlete tries to be involved in social affairs, such as charity, this will encourage other people to do charity work, and I always try to be that way. Well, of course, as an athlete, I'm expected to make the most of the possibilities of life, which it is. That is, I do not try to put myself in trouble at all and it meets my needs in the best and most logical way possible, but I never immerse myself in the constant consumption and change of the necessities of life…* |
| ***5*** | *(The interview with a young athlete inside an expensive tower. The place of the interview is where they say; The CEO of a famous sports team enters a sudden and intrusive night to catch the young and effective phenomenon of his team!)*  *What have you been doing since you woke up today? I was practicing today. We practiced at 10 o'clock in the morning, then I came home and had lunch and ... and then you (the interviewer) came.*  *What time did you wake up?* [*at 9 o'clock in the morning*](https://forum.wordreference.com/threads/at-9-oclock-in-the-morning-on-10-in-october.3692722/)  *When do you wake up on days when you do not exercise? Twelve am or one pm o'clock, noon summary!*  *How do your days usually spend? Tell us about your hobbies and hobbies: Usually if I do not practice at home. I leave the house very rarely.*  *Why are you so fugitive from the outside? I escape from traffic and crowds and these things. I prefer to be at home and comrades and comrades come together and say and laugh and these. This is my form of entertainment.*  *At the age of 21, you came from a team that barely had 200 fans, where sometimes 50,000 people might vote. How does it feel to have 50,000 people call a person by name? Well, it feels very sweet. One becomes one in a way. A good sort! But for a moment. His senses are momentary, they pass quickly. Then you come home and it's gone. No more, no more.*  *Many people think that you have no grief. You have? Very ... you said "very much" with sorrow!! What are your sorrows? It is personal. It is impossible to say ... why it is possible to be sad about the future. I'm afraid of the future. Now, as you say, everyone thinks that our situation is very good and that everything is going well for us and we are making money on the team ... I mean, you are not rowing? Not! It is not like that either. Not really. You go and sign a contract of 2 billion, what is the use? When there is no money, you can do a lot of art and it will take you 700 hands in the whole season.*  *In general, it seems that you really want to be in the eye and act in the first role. The first season that you just came to the team ...., you were interviewing the newspapers way byway! Somewhere you said tell me "Neymar Asia".*  *Well, the reason was that they called to interview me, and I did! Then I did not get the title. I did not say anywhere tell me Neymar! This was said by the fans, and I said in the interview that they said it*  *Are you one of those guys who care a lot about looks, clothes, and hairstyles? Yes, I am...*  *How many days do you go to the hairdresser? Once a week...*  *Do you just go to your own hairdresser or may you use other hairdressers as well? No, just my own hairdresser.*  *It is impossible to say exactly how much you spend on shoes and clothes per month. It has more or less. On average? It is about 7, 8, 10 million a month. Sometimes 15 million. It depends on what I want to buy. For example, one month I may buy a watch, which it becomes 10 million. Or, for example, two pairs of college can be bought for 7, 8 million.*  *Are you an open car? Do you change cars a lot? Yes, a lot, I always lose.*  *What are your machines now? Toyota GT red ... I believe my clothes and car should be great. I like to reach my type and appearance both in the game and off the field. Let everyone say whatever they want, there is no doubt that I have many enemies. In the first season, I came to Tehran and I played in a big team, thank God I did a great job and because of that, many people may not have eyes to see my success. I do not care if I am very sensitive and under the microscope more than others. The important thing is that I play good football and the fans have been satisfied with my game so far.*  *How much are you into movies and cinema? I watch movies but at home.*  *When was the last time you went to the cinema? I do not remember a long time ago.*  *What book? Do you read books? No, I do not read books.*  *What is your level of education? Diploma.*  *Did not have the opportunity to study more or did you not like it? No, I did not like it.* |
| ***6*** | *I think for an athlete like me, achieving balance should be the most important goal, now I mean balance, the athlete should be able to manage everything in his life together and in a balanced way. It is not easy to be the professional athlete that everyone expects from us. But it is not impossible and we must strive for it. I always try to be regular and on schedule as I work out. Outside of sports, it means that I should be regular even in shopping, entertainment, social relations, family relations, friendly relations, etc., and I should avoid excessive in these cases. If I follow these things, it will help my sports to grow, and in order to be able to strike a balance in my life, I must also use the help of capable people such as counselors and psychologists. Many athletes now spend a lot of time in cyberspace and even reduce their sleep and rest time to surf the internet. Is this kind of behavior, right? This is not true, but as an athlete, how should I be in cyberspace? First, I should have a cyberspace account and, if possible, hire someone to manage it, or I should be in cyberspace for a maximum of half an hour a day. Otherwise, if I spend a lot of time aimlessly in cyberspace, my athletic performance will definitely be disrupted. I have to prioritize my work according to their importance in order to achieve my goal, which is the development of skills and sports, and for this, I have to make rational decisions and even get a university education. I always try to treat everyone with respect, such as my coaches, teammates, even opponents, etc., because it makes me show a good image and others respect me. I believe that an athlete should do his best in exchange for a professional contract with a club, which will lead to better contracts with better conditions in the coming years and move to bigger teams.* |
| ***7*** | *What are the most important things in life for you? The most important thing for me in life is to achieve a lot of money and wealth, to have money and wealth, you have respect, if you do not have it, you will not have respect, even if you get a lot of honors in sports. Many say; our great footballers must also have high literacy and culture. They should read Shahnameh! He said that the national team player is no longer an ordinary person, so his culture should be of the same degree. His thoughts and behavior should be the same. Until he did not bring up filth and disgrace. I also understand that there is such a thing as "behavior". A player who is seen at a high level must be careful about his behavior. This is not a complicated thing to look for in books. Even now, almost none of our footballers go to read books. I agree with "doing the right thing", but it is not right to ask an athlete to be the best in his community. We have to see what the fans want from football. They want to score and win. The player of Esteghlal and Persepolis must know this well, otherwise, it would be useless to have a doctorate.*  *Is continuity and progress more important to you or making money? How much more do you think about earning in future elections? Of course, money is also very important to me. I try to have both. Both technical progress and high income.*  *What do you do if you have to choose? Suppose one side is a team that does not have a lot of money but takes you technically higher, and the other side is, for example, an Arab club standing with big money and low quality. I try to have both, but money is not something that can be passed over. How many years can I play? I will be 35 one day and I have to make good support for myself. Now you look at a certain player (player name). He played all these years in the Arab countries that you say do not have quality, instead, he guaranteed his future and now he lives in peace. What is wrong with becoming a multi-billionaire at the age of 35 and reigning for the rest of your life?*  *Do you prefer to become a multi-billionaire or have, for example, 100 national games? I definitely prefer the former. What if I do not have a secure future and want 100 national games? Do you think anyone respects ... all those games and national goals? No! He's credited with his billions of fortunes, not his games. .... Does it have fewer national games? Was he a low player? Where is he now?* |
| ***8*** | *I believe that the life of each of us, is made by ourselves. That is, we are responsible for our own way of life, and now we cannot live well without proper financial means. It is true that I am an athlete and I make good money from my contracts, but this is a fleeting period and I have to make the most of it. This means that I should try not to waste my financial resources, and on the contrary, I should invest by managing my living expenses in order to have good savings for my retirement. I try not to waste my money on buying and consuming supplies and things that I do not logically need. I mean, my purchases are always reasonable and I make decisions based on my needs. I choose friends who are like me, that is, they do not encourage me to waste my money, but even make me increase my financial ability by investing properly and earning money. I try not to comment too much on the political or social issues of the society because, firstly, I know that my comments and orientation will not have an effect on that issue, and secondly, it may cause me to miss some opportunities. Of course, I do not consider myself an opportunist, but I try not to get involved in issues that do not concern me. In order to be able to make the best decisions in my life, I try to get advice from trusted and expert people and make the best choice. Social respect and the expectations of the fans are important, but I am sure that if I do not have a good financial position, after the end of my sports career, I will not be able to meet even the simple needs of my family, and myself and in this case, even the respected I will not be real. So, I have to take advantage of opportunities and improve my financial life.* |
| ***9*** | *The national volleyball team of Iran is one of the teams that has made great progress in the past decades, but bad things happened to this team in the 2018 World Cup, so the performance of volleyball players was criticized by the media and volleyball fans in Iran. After the unfortunate events that took place for the Iranian national volleyball team, several experts made different comments. The following is an interview with one of the skilled players of the previous years of the Iranian national team, who now has a high educational background and has a lot of experience. The World Cup was a place where fans of the two volleyball fires saw no trace of the players' constant effort, and no results were as embarrassing as they were proud of their victories. No audience likes to give up their sweet morning sleep and watch events for which the reasons are not yet known, but the result was a defeat against the second and third world teams. There is a lot of talk about this team, from accusing players of being arrogant, making money into volleyball, the presence of people in a position for which they were not born, the inadequacy of their coach and assistants to hypotheses that are not allowed to be mentioned. According to this player with current experience and coach now, let me tell you a thousand reasons for technical and tactical problems. But if you ask me, what is hurting this team right now is a cultural challenge. To give an example, who spends all this time in cyberspace? This growth of our volleyball bubble has caused the reputation of children (volleyball players) to go beyond their capacity. The kids are all the best, but for example, go and ask what their average academic literacy is? Maybe lower than a diploma. This means that the whole world is full of money and volleyball, they say how much the lesson hurts us, and this view brings a thousand other kinds of problems. That is, none of the players in Iran goes out of the way of sports schools; And they grow very fast and suddenly find themselves in a situation for which they have not received any training. High reputation, high income, and dependence on the national team's performance on them make them not even consider themselves adhering to simple moral principles. Even though these athletes perform well in the game and consider themselves professional athletes, they are not professional athletes.* |
| ***10*** | *Interview with an athlete; Who was constantly questioned by the ethics committee in Iran for his special clothing and fashions, and therefore preferred to leave Iran for a while.*  *Considering what happened last season, can we say that you fled Iran? No, you cannot say such a thing about my decision because I had already made that decision. I told Mr. … and … in the middle of last season that I wanted to pursue football in Europe, and even Mr. … told me that it was the right decision and that you would surely succeed there. I can only say that those who should have known were on my way to...*  *I should not be in the Iranian media now because my job is somewhere else and my whole focus is on what I do. I came to Europe for a specific purpose and naturally, I only pursue my purpose. Thank God, in the new path I have found, everyone is satisfied with me, from our team fans to the players, coaches, and managers of the club, and this is the most important thing. Ask for the truth. I do not want to go back to the past. Now he wants this past to be terrible or wonderful. The fact is that my personal life is about myself and it is better to judge me on the playground while I am going through my own life right now and I am not thinking about anything. It's natural for me to go, and there are hills and pebbles along the way. But regardless of that, I go my main way to conquer a great peak.*  *If you go back in time, do you still choose yellow pants and weird types for your cover? I still wear yellow pants because I don't think yellow pants are a problem and it was my personal life. One can be two personalities and behave the same in appearance and dress and behave in a different model, but I do my job and I have my own taste. Now, if this taste is contrary to the general belief of society, it is also very important. No, because I have never commented on anyone's cover and no one has the right to talk about my cover. I have never told anyone that a dress is beautiful or ugly and I do not expect anyone to comment on this.*  *Do you think that the doors of the national team will open for you again with your performance? Sure; I feel that when you are on the right path, good things are behind it. Now one of the sons of Iran is on the national team instead of me, and he is trying to make Iran proud, and I wish success to all the goalkeepers of the national team.* |
| ***11*** | *In my opinion, an athlete should be professional in his/her life and should live in a professional style and context. All aspects of his life must be professional. Sleep, food, work, social behavior, life style must all be professional. Finally, professional means modern and advanced. It means specialized to look at the subject, you cannot be a professional when do not have professional tools and facilities. The professional sport and the professional athlete must move in parallel. Professional footballers, professional leagues, professional sponsors and all must be provided. When you grow up (fame), surely one of the factors that can help a person to progress further in life is the presence of a counselor. Good advisors would be helpful in behavior and performance, mental and psychological, nutrition, dressing, dealing with the media, interviewing, appearance type, how to spend the money that the athlete earns and etc. Unfortunately, our football is called professional, but there are many problems. Our athletes eat fast food (Fatty foods and soft drinks) six days a week because they are single. I think our athletes live traditional-modern and integrate everything. Because my brother chose football, so I go to football (influenced by relatives). After becoming a professional and going to university and progressing, I became a little more modern. Many of us have to do something by force so I think more regular coach will do better. Athletes are role models. Unfortunately, books do not fit in our society. Reading books is important, but unfortunately, it is not noticed at all. A high percentage of our athletes are not even high education and do not have any studies. Our sports magazines do not carry a culture that leads to an increase in athletes' literacy and knowledge, but only cover events and negative points. Culture is a missing topic in sports. The appropriate age for training athletes is 12-18 years. The promotion of football and culture building is not done even in football schools. And this is where family history and background and family origins are more important. The athlete, despite being good at technical discussion, will be drawn in inappropriate ways. The best way is to educate these people, at least about living.*  *There should be no coercion and one should accept everything with interest. For example, in the NBA, an athlete must enter the league from university, which shows the need for university education.*  *First, strong disciplinary regulations can be enforced for athletes through contracts.* |
| ***12*** | *We do not have a professional activity (plan) in our athletes at all, so this spreads to all disciplines and most of our athletes do not have professional behavior, do not have a professional life, and only follow the conditions that the teams have define in camp for them such as nutrition, sleep and other issues. Unfortunately, this defect exists in all sports in Iran, especially football and futsal. We only provide professional conditions for them on the night before of match. The Professional (lifestyle) means that in return for the money received, obligations are created for her/him that she/he must fulfill about not only training but also rest (leisure) and other activities.*  *There are several categories of athletes' lifestyles:*  *A group that suddenly received a lot of money if they were not ready to receive it at all. The luxury car and the attention of the community, etc., will definitely get in trouble. They have reached money over time and they have adapted to money. These people have grown up logically, and so are successful. When they got the money, they set up a business with it and even had innovation and creativity so that they became a rich person. Cultural consumption (for study purposes) in athletes is below zero.*  *The percentage of study should be increased. Unfortunately, the family, schools or the federation does nothing. Athletes often tend to watch movies and even vulgar ones. Our biggest problem is that our clubs are completely sports and do nothing in the cultural sector. Coercion in European clubs leads to the internalization of professionalism. The phone should not ring at all (during training)*  *The Iranian athlete is strongly dependent on the family and is strongly dependent on the mother from any class.*  *To institutionalize these cases, one must start from the* ***playing*** *contract. Monitoring the implementation of the contract is very important, and if he/she was careful in controlling and monitoring and did not differentiate between individuals, it will surely become internalized in the future and become a professional behavior. Athletes spend most of their time with friends, especially the opposite sex, and spend less time with family.* |
| ***13*** | *The courses we take for coaching talk very limited about the lifestyle of athletes. But in general, three styles in the life of athletes can be distinguished:*  *Professional: A style defined. They have a detailed plan for nutrition, rest, exercise, leisure, private life.*  *Traditional: They have learned things from the past but they do not want to change it and they are dependent on it.*  *Normal (everyday): has no specific schedule, is tired, sleeps, does not take care of himself and has a life influenced by the environment and friends. Affected by his own symptoms and those of society.*  *Professionals have consultants in various fields. We call these people professionals*  *And the criterion is not to take money or not.*  *Among teenagers and young adults, we have people who are looking for a professional program while they do not currently have a contract or receive money.*  *In developed countries, these things are taught from the academy. Even etiquette.*  *Because they say (believe), athletes will have two important indicators in the future: Riches and Celebrity. And for both of these it must be trained*  *In some professional countries, there are chapters to teach economic and various issues to famous and popular people*  *Iranian athletes have neither financial expertise nor a good view of financial matters. Most of them do not even have a consultant.*  *They are often in the business of changing phones and cars, but some also buy houses. Some are also looking to invest. Most athletes do not have a view on financial issues and get into trouble. After the end of the player's term, some leave and do not last. They make no change and come back.*  *Some people stay and change despite the difficulties, and after returning, the changes can be seen in their various issues. These are few in number.*  *It goes back to the first three styles:*  *Everyday life is not purposeful; they end up thinking about the next season and where to go.*  *But professionals have a farther perspective and are thinking about the future.* |
| ***14*** | *An elite athlete is someone who acts at the highest level in our country.*  *I played in the first level of the country for a few years and now I am coaching. The most important factor in my success was that I loved football and futsal and it was the main and first priority of my life, I thought about sports in any situation and that is why I endured difficult conditions.*  *I was looking for exercise with my heart and this made me resistant to hardships.*  *I mostly tried to have discipline in my work.*  *When I was playing, the most important thing I was involved with was what the coach thinks of me?!*  *What is the coach's attitude towards me?*  *My concern was not to have a negative attitude towards me, either behaviorally or technically, and I tried to achieve this with the normal dressing, the right behavior, the so much effort, and I did not play a role. It was really important to me. I did not play a role and I really respected it.*  *Correct attitude and behavior towards the opposing team and the opponent.*  *I never thought of succeeding in the wrong way.*  *I never acted outside the framework and acted according to the defined principles of the field (behavioral, technical, moral, etc.).*  *I believe that everything has a right and logical way and I have always tried to follow it.*  *My first priority was not to win*  *And healthy exercise and proper work were very important to me.*  *In addition to technical issues from an early age, other issues should be taught to the athlete such as how to talk, how to communicate, foresight, economic issues and ....*  *Money has entered our sport, but it has not been spent in the right places, it has been spent in the waste places*  *The athlete should be taught two different sessions a week.*  *Our athletes have weaknesses and they want to cover their weaknesses and show that they are great people by spending a lot of money indiscriminately. (Become famous instead of popular)*  *I have always tried to be honest in my relationships with different people, and honesty has been one of the most important principles in my life.*  *Regarding elite athlete relationships*  *Family - student - teammate - coach - community - media - supervisor and ....*  *I have always tried to be honest in my relationships.*  *The one who commits dishonesty wants to achieve benefits through this dishonesty without enough effort*  *Studying and paying attention to cultural issues is essential for athletes.*  *Study leads to awareness and will change the insight and eventually the right behavior will occur in the person and this in turn will cause other people to model appropriate behaviors.*  *These are more important than seeing the car. Even the car can lead to social inequalities. Special attention to football schools in all disciplines. Pay attention to children in golden ages.*  *A capable coach does not work due to the lack of sufficient facilities and income at low levels, and this opens the field for people who may not even be morally and behaviorally honest.*  *The main problems that occur in the lives of high-level athletes are because our athlete has not risen systematically and has not received enough training.* |
| ***15*** | *Most of the issues are related to personal lifestyle and can be very different between different athletes. Some athletes treat different issues of normal life, but others like to have a certain style and be prominent.*  *Overall, what matters to the elite athlete is the championship. He/she has to live in such a way that has the best performance in sports. The focus is on exercise and it affects all of life.*  *An elite athlete can not travel like other ordinary people in society, because if he is separated from training for two or three days, he will suffer from a decline in athletic performance and will have to redouble his efforts to compensate. The athlete should be fined if he/she stays up late and walks around the city.*  *In our country, most of the ways and lifestyles are personal unless the player is in the camp. The most important thing for athletes is to prepare for training. Just as a training session is important for the athlete, so is recovery and rest after it. Communication with the media and being finger pointing is very important and can affect his performance. Individual tastes are very influential in the choice of fashion and clothing and other things, but I do not think it will affect performance.*  *Most famous players are in a good financial position, in other words, they sign good contracts, but not in the face of martial arts, wrestling and karate athletes, and they are in financial difficulties.*  *But we see that this financial difference has not had much effect on their sporting success, and individual athletes have been more successful. Just as humans are very different, there are many differences between athletes. The athlete must be mentally prepared for the new position.*  *The margins of European athletes are much smaller than our athletes. Our athlete is suddenly placed in a new position without preparation, and if he does not have the capacity, he will run into problems. Ideally, systematic planning should be done so that athletes are not drawn to their personal tastes in any direction. Because sports clubs need the player's performance, they do not do a lot of hard work in the cultural direction, and even in some cases, due to the technical need for the player, his immoral actions are not dealt with and ignored even sports federations.* |
| ***16*** | *People have different styles. Some people have no plan and spend incalculably, but others, despite earning little money, spend with a plan. It can be said that Iranian athletes, despite a good income, stay at the lowest costs after the end of the sports period. The economic lifestyle of Iranian athletes is very abandoned and disorderly and does not have a good condition. If we ask 10 athletes about economic issues, we cannot find any particular style among them because it is very chaotic and has a messy state….*  *…. There are some athletes, who, with the first money receive, quickly go for their clothes, mobile phones and cars, but some of them also buy house or invest in other work. Therefore, athletes have fundamental differences in terms of economic style. Of course, these cases depend on various factors*  *The first money I got myself went and got shoes and clothes, but it can be said that these things depend on many other variables (family history, shortcomings, etc.). For example, views on self-insurance, life insurance, etc., making a lasting investment or wasting financial resources and income…*  *...Athlete etiquette is very important and how to communicate with people. Communication to media, family members, spouse, playmate, community, reporter, club managers, coach and referee…,*  *In terms of how to use the tools and facilities or the type of consumption, some are consumed or consumer-oriented, but some consume well and are frugal…, Some athletes have been productive (in fact, they have had a productive view: physical production is not necessarily involved and includes useful behaviors). For example, one likes to do something useful, and when enters a place where help is needed, she/he immediately helps, but some just sit and wait for something to be done for them….*  *…. Having a professional view of sports is very important for athletes and should be considered as a continuum. For example, there are people who are looking for a suitable club. Usually, these things are taught in the world, but because we do not have education, we have different methods.*  *…. The professional lifestyle is not just about the financial aspect, but should include all aspects of an athlete's life. Usually, these things are taught in the world, but in Iran, because we do not have education, we have different styles and moods, and the players who go abroad learn these things and teach them to others as well.* |
| ***17*** | *…For athletes who are proud, their love for the people and the country must be strengthened, and the level of patriotism and nationalism of these people is an important indicator in life…., Due to the role models of athletes in society, behavioral issues should be taught to them and desirable behaviors should be reinforced.*  *…Scheduling is very important in different cases of athlete’s life as well as Individual hygiene and appearance….*  *…Travel etiquette and presence in a foreign country is very important …, Elite athletes must be the foundation of etiquette. Also, etiquette is extremely important in communication with family, coach, supervisor, playmate, …, and in addition to communication, this communication should be respectful and based on respect. In relation to the opposite sex, does the athlete follow national culture and custom or act according to Western patterns? Does he follow the red lines in his communication?*  *…A professional athlete should follow the etiquette of professionalism and not have an amateur personality, but many of our athletes are unfortunately amateurs. Professional behaviors in training, competition and...,*  *Self-care and self-management are very important for athletes, due to the various injuries that threaten young people today, especially athletes (due to high incomes and fame), proper education should be given to athletes… Self-care and self-management must be strengthened…,*  *…An athlete must maintain his public relation. Experience has shown that in our country, athletes become nationally popular figures and even go down in history as being with the people and not being separated from the people. Being with people can show in the neighborhood and area where athletes are living…,*  *…Due to the high income during the playing period, economic skills and investment abilities should be strengthened in elite athletes, and these people should not be just looking for luxuries…,*  *…Some athletes go to doping, which can be called “cotton Pahlavān” (a Persian term meaning hollow or fake champion). Efforts should be made to condemn doping as ugly. Adherence to pure sport is important…,*  *…Familiarity with new technologies such as Internet skills, virtual networks, etc. is necessary, so that these people may not be abused, and of course, it is necessary that the amount of their use is reasonable and based on a certain rule…,*  *The growth of athletes must be balanced, and this shows the need to pay attention to cultural and social issues, these people must attach great importance to study (although the newspaper), as well as specialized study in their fields. Athletes must be trained to be able to withstand multiple social injuries. It can even be said that due to the popularity of athletes, some people are in ambush.* |
| ***18*** | *Given the breadth of lifestyle, it is better to study several indicators in order to study more deeply, increasing the probability of athletes responding. You can also consider all the issues that are considered as indicators and examples of lifestyle for athletes, but due to the special characteristics of athletes and their issues in society, some things that are more important in the lives of these people would be as follow.*  *In my opinion, you can consider three main indicators for the lifestyle of elite athletes:*  *What is the economic style of athletes? Refers to have planning in economics issues, and an athlete spends money without predicting the future. Unfortunately, many athletes in our country have no plans for how to spend and will run into problems in the future. Therefore, it is necessary to strengthen economic management in these people. Many athletes use unnecessary spending as a tool to express their personality…,*  *…Given the role models of athletes in society, the moral and behavioral dimension of these people is very important. Positive patterns should be strengthened in these people so that others can imitate the positive points of athletes by observing desirable behaviors, not negative behaviors (for example, excessive luxury, etc.).*  *When discussing ethics, we can even consider an athlete's perspective and behavior regarding drug use. An athlete, who adheres to ethics, considers inappropriate to use of such substances and any unconventional method for achieve the goals, and it is natural that he will not go for it…, Mental health is very important for athletes and mental health means the same as spiritual lifestyle…,*  *The communication style of people, especially athletes, is important, and unfortunately, most of these people now spend time on mobile social networks, so a mechanism must be adopted to reduce the negative consequences of this type of communication.* |
| ***19*** | *Lifestyles are different for different people and therefore different for different athletes, despite the fact that these people have one thing in common, which is championship sports. Some athletes think that they should exercise well and become champions. However, others like to study in addition to sports. Some people like to have a managerial position during championship sports. Alternatively, some athletes like political situations. Some persons who only exercise do not care about other things so do whatever they want; they are dressing as they please. They do not have many good behaviors and do not have a so-called vision for the future. Alternatively, some have a plan and live with a plan. They have a purposeful and regular lifestyle. They spend enough time studying and doing other things. Instead of social networking, they focus on new topics and even are acquainted with the lifestyle of other athletes in other countries. In general, the lifestyle of athletes is very different from ordinary people. They cannot have the entertainment that people have. For example, a famous athlete does not lick ice cream on the street. Their entertainment becomes special and closed. And less likely to appear in public settings such as amusement parks…. The type of food is important and who the athlete associates with or not…. The choice of friends and comrades is very important and have limitations…. The elite athlete considers it very important to be noticed and approved.*  *There are two main reasons why some athletes go for doping. They feel that they reach the goal sooner and better with doping. They think that all the athletes in the world consume and they are behind.*  *In fact, the elite athletes see doping, as a technology, which they feel has not been achieved. So they go for doping. This cultural misconception has arisen.*  *Economic issues come first for ordinary people as well as for elite athletes*  *Some elite athletes invest in a pizza restaurants or cafeteria and are willing to give hookahs to people for profit.*  *Many athletes are motivated to win the same financial issues, and some who do not improve their financial situation by giving up the sport or their performance declines and move on to other jobs. Therefore, good economic conditions can lead to better performance.*  *Sports and athletes are one of the most important cultural categories that can help society. If 1000 sociologists or clerics are brought to TV and advertised and told to dress like this or have a hairstyle like this, the youth and teenagers will not pay attention, but as soon as an athlete does something, he will become a role model immediately.*  *Unfortunately, no special work has been done on the cultural issues of the athletes, and their cultural issues are very beginner.*  *We only asked an athlete for a performance, we only asked for a medal, we only asked for a goal, and we do not pay attention to his morals until he makes a mistake.*  *Personal style is important in cultural consumption issues and people are different.*  *The athletes of team sports often have group fun such as football and volleyball*  *However, the athletes of individual sports are more isolated and spend more time alone.*  *Someone who does professional sports should have a professional life.*  *No one can do professional sports and live an amateur life. Professional life has its own framework. The first part is just the exercises. Good rest, nutrition, leisure and recreational activities are very important.*  *There is no specific lifestyle for athletes, and it has even happened a lot that athletes are dragged from the national team camps in inappropriate ways and disappear.*  *For example, when I went to the national team camp, I looked at the old people to see how they live and I was looking for a model that was not there!* |
| ***20*** | *First, I define an elite athlete. In my opinion, athletes who train regularly (professionally) at the national level, or at least in the top leagues of their sport or at the semi-professional level. These people have a contract and they have to participate in training and competitions according to the contract and they are paid, I consider these people as elite athletes.*  *Characteristics of the lifestyle of these people*  *Two types of characteristics can be considered for athletes: sports characteristics and non-sports characteristics*  *1. Sports characteristics: What style do they have in sports? Each sport has its own style. Footballers, basketball players and ...*  *The characteristics of any sport can be one of the components of athletes' lifestyles, but these characteristics are like religion or gender, in other words, people who accept activity in one discipline must observe them.*  *A person who plays football must follow the relevant principles. Example*  *Soccer lifestyle, martial lifestyle, wrestler lifestyle. We now see that many wrestlers have studied and been educated.*  *Sports characteristics can also be divided into two parts:*  *Pattern styles related to each specific discipline (general for all athletes in that discipline)*  *Non-standard styles related to each person (for example, the professional style of each athlete). The most famous professional athletes in the world have a special style for themselves. A professional athlete must comply with the framework and must observe them (whether they like it or not), for example, he must sleep at ten o'clock at night and observe these things in the camps.*  *In leading countries, athletes must follow strict regulations and be under the supervision of the club.*  *Variables such as regular sleep, type of nutrition, means of transportation to training, etc. in Iran are mainly at the disposal of the athlete, unlike foreign countries.*  *We have the same things in sports. Foreign clubs even plan to feed their athletes, but this is not the case in Iran, and usually the athlete craves any food he wants, regardless of whether this food is suitable for him or for this sport or not?*  *When we were playing, we ate chelokbab berg (a kind of traditional Iranian food) before the match and did not pay attention to how much energy our digestion of this food takes while playing. That is, our lifestyle was that we thought this food was good for us and even had a certain prestige. We considered this to be the highest (most appropriate) thing.*  *In the case of athlete sleep, we need frameworks for this, and we need to think and react to it for our athletes.*  *2. non-sporting characteristics of athletes' lifestyle*  *The issue of worship: Some people, although they are Muslims, are not very constrained and do not observe (the prayer is made up), but it is very important to them: issues related to religious duties.*  *Cleanliness and hygiene: Regular hand washing, regular clothing and shoes, shaving, waxed shoes, ironed clothing, tidy hair and body appearance are very important to some people and they are tidy but others are not.*  *Some people shave their beards every day, but one may trim their beard, but some do not shave or take care of their beard.*  *Being tidy in school and living, working hours, having a regular personal and work schedule, taking notes and recording all things, especially work, being committed to tasks and being tidy in appointments and activities*  *Does he work hours, does he write his schedule or not? Some people promise and of course do not pay attention to it and do not accept any responsibility.*  *I say everything based on Glasser's choice theory approach. He believes that we choose everything ourselves. This choice is based on our inner characteristics.*  *We all choose our lifestyles based on our inner characteristics.*  *For example, Freud believes that in the first 5-6 years of a character's life is formed and in the rest of our lives we see its manifestations.*  *We all choose. Select the type of film, the type of music, the type of cultural product. This choice is not necessarily according to taste and one may want to experience other tastes as well.*  *We have different worldviews for a number of people. We have one world but different worldviews.*  *Taste is one of the factors influencing the choice but it is not decisive, for example, personality can also be influential.*  *People get more things that are in line with their mood. For example, if I have a happy mood, I would like to see comic film rather than a classical theater.*  *Goal setting or foresight is very important for athletes and people are different in this regard. I was a member of the national team when I was a student, but I left the national team because of the lesson, which surprised everyone and they said that is it possible for someone to leave the national team because of the lesson!!!!*  *The coach is very influential in foresight.*  *Putting the national team aside for lessons*  *Or focus on sports until lessons*  *Or enter other jobs*  *Many are not forward-thinking, Americans try to make their athletes forward-thinking, that is, while participating in professional sports, they participate in various specialized courses (marketing, coaching, costume design, etc.).*  *Drugs can be used for recreation or as a result of the pressures of life.*  *Doping is precisely related to performance, or occurs to look good and fit and to be muscular or to enhance performance.*  *We try to use all the factors that enhance our performance, and even an athlete may use unconventional methods.*  *Recreation and leisure is one of the most important issues in the lives of people today, including athletes, and athletes may turn to drugs for fun.*  *The way of looking at life include being result-oriented and process-oriented. Result-oriented can affect the consumption of energy substances. But process-oriented people pay attention to the path and move forward step by step. Result-oriented: One only care about the result and tries to get results at any cost.*  *One goes to the process and therefore steps by step.* |
