# Supplementary material for: Elite athletes’ lifestyles: Consumerism to professionalism
Source: PLoS One. 2022 Sep 26;17(9):e0269287. doi: 10.1371/journal.pone.0269287 (PMC9512170; doi:10.1371/journal.pone.0269287)
Supplement: Attachment — Submitted filename: Elite Athletes Lifestyles Consumerism to Professionalism.docx [file pone.0269287.s002.docx]

Elite Athletes' Lifestyles: Consumerism to Professionalism

Thank you to the author(s) for submitting their manuscript to the Plos One Journal. Please see the page-by-page comments below. There seemed to be a couple of main issues that, if addressed, I think would enhance the research. For one, I question the need and purpose of the paper in relation to theoretical and practical implications of the research. That is, why there is a need to study lifestyles, what problem does it help with better understanding, and how the knowledge will be used and by who. Additionally, I believe the theoretical rationale for why the reviewed theories were not used, can be strengthened. I hope the author(s) are able to reflect on the comments and feedback in their future iterations of their paper.

Page 6: Paragraph above table 1: Could the author(s) also unfold concisely the studies that have used athletes and what to further explore in this research that the current paper/research addresses?

Page 7: It is believed paragraph: In another set of studies sentence and the remaining components of this paragraph: See previous comment as it seems here the athlete studies are being addressed. Could the author(s)articulate and strengthen the differentiation or distinction between the points in the paragraph above the table and the research included below?

Page 8: So far paragraph: Therefore, if lifestyle: The research prior could be better organized and tighter around these points. As such, was the health orientated lifestyle addressed.

Page 8: I am still unclear on how the reviewed theories could not be used to comprehensively explore the different aspects of their life—on and off the field. I think this rationale can be strengthened. As a suggestion, the author(s) might add a bit more of a critique in the beginning aspects of the paper where the theories are reviewed.

Page 9-11: Methods: could the author(s) highlight their epistemological and ontological assumptions/paradigmatic of their research.

Page 13: The components of the findings here might be better served as data collection and analysis procedures sections within the methods. Further, an added component on quality research standards would be appropriate.

Smith, B., & McGannon, K. R. (2018). Developing rigor in qualitative research: Problems and opportunities within sport and exercise psychology. *International Review of Sport and Exercise Psychology, 11*, 101-212.

Smith, B., Sparkes, A., & Caddick, N. (2014). Judging qualitative research. In L. Nelson, R. Groom, & P. Potrac (Eds.), *Research methods in sports coaching* (pp*.* 192–202). Routledge.

Sparkes, A. C., & Smith, B. (2014). *Qualitative research methods in sport, exercise and health: From process to product*. Routledge.

Page 14: There is a need to organize and describe these codes across the participants. Descriptions in what their deeper meaning are into a coherent description of the groups of open coding would be useful. See previous comment as it may open space for this.

Page 15: How were the codes/groupings in phase one used or informs phases 2?

Page 17: Style 2: Individuals in this group: Individuals in this group seem to lend itself to the idea that only part of athletes discussed this component. But, couldn’t there be integration across these themes/styles that could be involved in different aspects of the life, for different things, etc. Please clarify.

Page 19: Discussion: Some of the key points seem to run together in the discussion. Further, it would be useful to compare and contrast the theory generated from the research and Bourdieu. Some of the discussion involves articulating Bourdieu’s theory in relation to the findings, without as much consideration for the theory generated from the research. Based on this, I again, question, why couldn’t Bourdieu have been used in this research as I had indicated in a couple of other comments. Nonetheless, it would be useful to critique and compare and contrast Bourdieu to the findings. A final recommendation would be to include practical implications of the research findings. That is, who does the knowledge from this research address, what problem does it help solve or gain a better understanding towards, and how does this knowledge get used in practical application. Perhaps, there could be some indication of this in the introduction section from which the discussion would tie together in response the new knowledge generated from the findings.
