# Supplementary material for: Elite athletes’ lifestyles: Consumerism to professionalism
Source: PLoS One. 2022 Sep 26;17(9):e0269287. doi: 10.1371/journal.pone.0269287 (PMC9512170; doi:10.1371/journal.pone.0269287)
Supplement: Attachment — Submitted filename: Response to Reviewers.docx [file pone.0269287.s003.docx]

Dear Editor-in-chief/Reviewers

We want to express our gratitude for creating the opportunity to improve the quality of our manuscript. It was very gratifying for us to get the favorable opinion of the reviewers in the first stage. We also appreciate the detailed comments of the reviewers, and we believe that these comments and possible future comments can significantly improve the quality of our manuscript. We highlighted the changes in the manuscript with colored text as follows.

1) In response to the first reviewer's comment about the quality and content of the pictures in the text, we redesigned the main model of the paper in Photoshop in compliance with PLOS ONE's requirements.

2) We explained more in the introduction why the reviewed theories were ineffective for elite athletes. Additionally, we highlighted the differences between the above and below studies of table 1.

3) In the method section, we described our paradigmatic assumptions. Here, we used the valuable sources recommended by the reviewer.

4) We added a section on research quality standards in the method section and separated data collection and analysis procedures.

5) To better organize the codes across participants, a column has been added to table 3, and table 4 has been added. Tables 3 and 4 show the codes generated by each participant.

6) For the section on Typology of Elite Athletes' Lifestyles, we have added an explanation about using the first phase codes in phase 2.

7) Concerning style 2 (easygoing style), we can say that easygoing athletes may be placed in other styles since they make immediate decisions and do what is easy for them. One day they might be a consumerist, and another day they might prefer social activities. As a result, they were assigned a particular style (easygoing) because they lacked the necessary stability in other styles.

8) We have further explained our study's differences from Bourdieu's theory in the Discussion section. Additionally, we discussed the possible applications of our findings. Of course, we must note that our findings were based on a qualitative study, and the possibility of generalizing the results is weak.

We hope to satisfy the editor-in-chief and the reviewers, although the authors would be happy to announce their readiness for possible future revision.

Best wishes

Research team
